# Human Infection with *Burkholderia thailandensis*, China, 2013

**DOI:** 10.3201/eid2405.180238

**Published:** 2018-05

**Authors:** David A.B. Dance, Derek Sarovich, Erin P. Price, Direk Limmathurotsakul, Bart J. Currie

**Affiliations:** Lao-Oxford-Mahosot Hospital-Wellcome Trust Research Unit, Vientiane, Laos (D.A.B. Dance);; Centre for Tropical Medicine and Global Health, University of Oxford, Oxford, UK (D.A.B. Dance);; London School of Hygiene and Tropical Medicine, London, UK (D.A.B. Dance);; University of the Sunshine Coast, Sippy Downs, Queensland, Australia (D. Sarovich, E.P. Price);; Mahidol-Oxford Research Unit, Bangkok, Thailand (D. Limmathurotsakul);; Royal Darwin Hospital and Menzies School of Health Research, Darwin, Northern Territory, Australia (B.J. Currie)

**Keywords:** melioidosis, pseudomallei, Burkholderia thailandensis, China, bacteria

**To the Editor:** We read with interest the research letter from Chang et al. (*1*). To have such severe clinical disease attributed to *Burkholderia thailandensis* infection published in peer-reviewed literature is of major significance to the research community, especially given the biosecurity aspects regarding melioidosis. We are writing, however, because we have serious doubts about the identity of the organism described.

The clinical features of the case are typical of septicemic melioidosis with pulmonary involvement. The pictures of the colonies in the technical appendix look very similar to *B. pseudomallei*, with which we have extensive experience in both Australia and Southeast Asia over the past 30 years. This identification was the most likely suggested by the phenotypic tests used. Furthermore, the virulence of the strain in mice bore more resemblance to that of *B. pseudomallei* than *B. thailandensis*, and the strain contained putative virulence determinants not normally found in *B. thailandensis*. Species identification thus appears to rest on arabinose assimilation and 16S rDNA sequence. Assimilation tests are notoriously difficult to read, and without knowledge of the 16S rDNA primers or sequence region of comparison, it is plausible that a lack of resolution between *B. pseudomallei* and *B. thailandensis* has led to incorrect species attribution.

We therefore believe that there is insufficient evidence to prove that this case was caused by *B. thailandensis* and that the presented data suggest that this isolate was, in fact, *B. pseudomallei*. We are always happy to advise colleagues about the investigation and management of possible cases of melioidosis, but in these circumstances, we felt that it was necessary to place our concerns on record.
